# Response assessment of NovoTTF-100A versus best physician's choice chemotherapy in recurrent glioblastoma

**DOI:** 10.1002/cam4.210

**Published:** 2014-02-14

**Authors:** Eric T Wong, Edwin Lok, Kenneth D Swanson, Shiva Gautam, Herbert H Engelhard, Frank Lieberman, Sophie Taillibert, Zvi Ram, John L Villano

**Affiliations:** 1 Brain Tumor Center and Neuro-Oncology Unit, Department of Neurology, Beth Israel Deaconess Medical CenterBoston, Massachusetts; 2 Division of Biostatistics, Department of Medicine, Beth Israel Deaconess Medical CenterBoston, Massachusetts; 3 Departments of Neurological Surgery and Bioengineering, University of Illinois Hospital and Health Sciences SystemChicago, Illinois; 4 Department of Neurosurgery, University of Pittsburgh Medical CenterPittsburgh, Pennsylvania; 5 Fédération de Neurologie 2, Groupe Hospitalier Pitié-Salpétrière, Université Pierre et Marie Curie Paris VIParis, France; 6 Department of Neurosurgery, Tel Aviv Medical CenterTel Aviv, Israel; 7 Markey Cancer Center, University of Kentucky Health CareLexington, Kentucky

**Keywords:** NovoTTF-100A, recurrent glioblastoma, response

## Abstract

The NovoTTF-100A device emits frequency-tuned alternating electric fields that interfere with tumor cell mitosis. In phase III trial for recurrent glioblastomas, NovoTTF-100A was shown to have equivalent efficacy and less toxicity when compared to Best Physician's Choice (BPC) chemotherapy. We analyzed the characteristics of responders and nonresponders in both cohorts to determine the characteristics of response and potential predictive factors. Tumor response and progression were determined by Macdonald criteria. Time to response, response duration, progression-free survival (PFS) ± Simon–Makuch correction, overall survival (OS), prognostic factors, and relative hazard rates were compared between responders and nonresponders. Median response duration was 7.3 versus 5.6 months for NovoTTF-100A and BPC chemotherapy, respectively (*P* = 0.0009). Five of 14 NovoTTF-100A responders but none of seven BPC responders had prior low-grade histology. Mean cumulative dexamethasone dose was 35.9 mg for responders versus 485.6 mg for nonresponders in the NovoTTF-100A cohort (*P* < 0.0001). Hazard analysis showed delayed tumor progression in responders compared to nonresponders. Simon–Makuch-adjusted PFS was longer in responders than in nonresponders treated with NovoTTF-100A (*P* = 0.0007) or BPC chemotherapy (*P* = 0.0222). Median OS was longer for responders than nonresponders treated with NovoTTF-100A (*P* < 0.0001) and BPC chemotherapy (*P* = 0.0235). Pearson analysis showed strong correlation between response and OS in NovoTTF-100A (*P* = 0.0002) but not in BPC cohort (*P* = 0.2900). Our results indicate that the response characteristics favor NovoTTF-100A and data on prior low-grade histology and dexamethasone suggest potential genetic and epigenetic determinants of NovoTTF-100A response.

## Introduction

Patients with recurrent glioblastoma have poor prognosis. Objective response rates to alkylating chemotherapy are low, ranging 5–8% 1,2. Although bevacizumab has a remarkably high response rate of 25–60% 3, its ability to prolong the overall survival (OS) of patients in the recurrence setting is marginal and it still awaits testing in a randomized clinical trial 4,5. Those who failed bevacizumab are unlikely to respond to subsequent therapy 6,7. Therefore, new and innovative therapies are needed for recurrent glioblastoma.

The NovoTTF-100A device is a novel cancer treatment that delivers low-intensity, intermediate frequency (200 kHz) tumor treating electric fields (TTFields) via transducer arrays applied onto the scalp. TTFields disrupt glioblastoma cells during mitosis, resulting in apoptosis, aneuploidy, asymmetric chromosome segregation, and defects in centrioles and mitotic spindles 8–10. In a phase III trial for recurrent glioblastoma, this device has been shown to have equivalent efficacy when compared to conventional chemotherapies, including bevacizumab 11. Notably, the NovoTTF-100A cohort had more responders than the Best Physician's Choice (BPC) chemotherapy cohort 11,12 and NovoTTF-100A responders may offer insights into the mechanisms of action of TTFields on glioblastoma. Therefore, we undertake this post hoc analysis on the characteristics between responders in the NovoTTF-100A and BPC cohorts, as well as differences between responders and nonresponders within each cohort.

## Patients and Methods

### Patients

**Figure 1:**
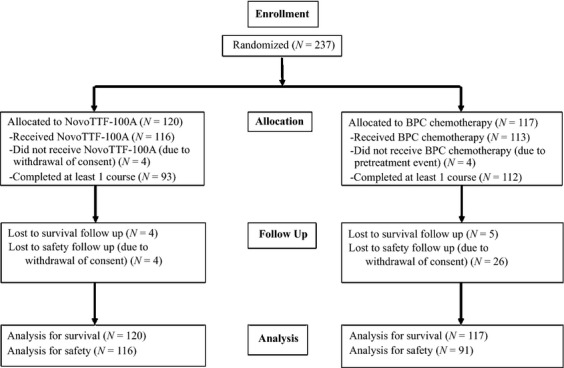
CONSORT diagram.

The conduct and the overall results of the pivotal phase III trial were previously published 11 and outlined in the CONSORT diagram (Fig. 1). Tumor response and progression were determined according to Macdonald criteria 13 and confirmed by independent radiology review.

### Statistical analysis

The corresponding author has full access to the data and is responsible for the outcome of analysis. Kaplan–Meier distributions 14 were generated using the R statistical package (http://www.r-project.org). The median, 95% confidence interval (95% CI), and *P* values were computed for time to response and response duration for responders in both NovoTTF-100A and BPC chemotherapy cohorts. Prognostic factors were compared between groups using Wilcoxon rank-sum test.

To examine whether NovoTTF-100A had a greater or weaker efficacy over BPC chemotherapy, we computed the relative density of hazard rates for responders and nonresponders to determine an increasing or decreasing rate of tumor progression 15,16. Plots of hazard rate density as a function of time to tumor progression were generated using R.

OS and progression-free survival (PFS) between responders and nonresponders were analyzed using Kaplan–Meier statistics 14. Additional PFS analysis was done to minimize potential bias in the responder population by introducing the Simon–Makuch correction 17,18. This was done by adding the median time to response to both responders' response duration and nonresponders' time to progression, followed by derivation of Kaplan–Meier distributions. The median PFS and 95% CI were computed in the adjusted groups and independence was tested by chi-squared statistics.

The distribution of OS was also compared to time to response and response duration. Linear regression was fitted to determine a one-to-one relationship between the two time intervals and the *r*^2^ value was computed. Pearson rank coefficient was computed to determine the strengths of the correlation. A scatter plot of the two time intervals was generated in R and independence was tested by chi-squared statistics.

## Results

### Responder characteristics

**Figure 2:**
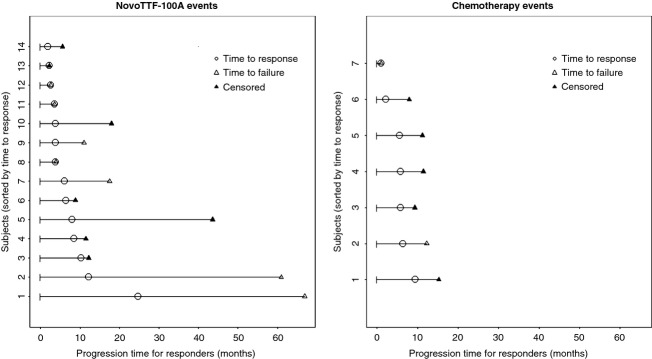
Event chart for responders in the NovoTTF-100A and BPC chemotherapy cohorts. Each line represents a single patient and patients are sorted according to the time to response. Transition between states, that is response and failure, are indicated by the corresponding symbols represented on the time line. BPC, Best Physician's Choice.

**Figure 3:**
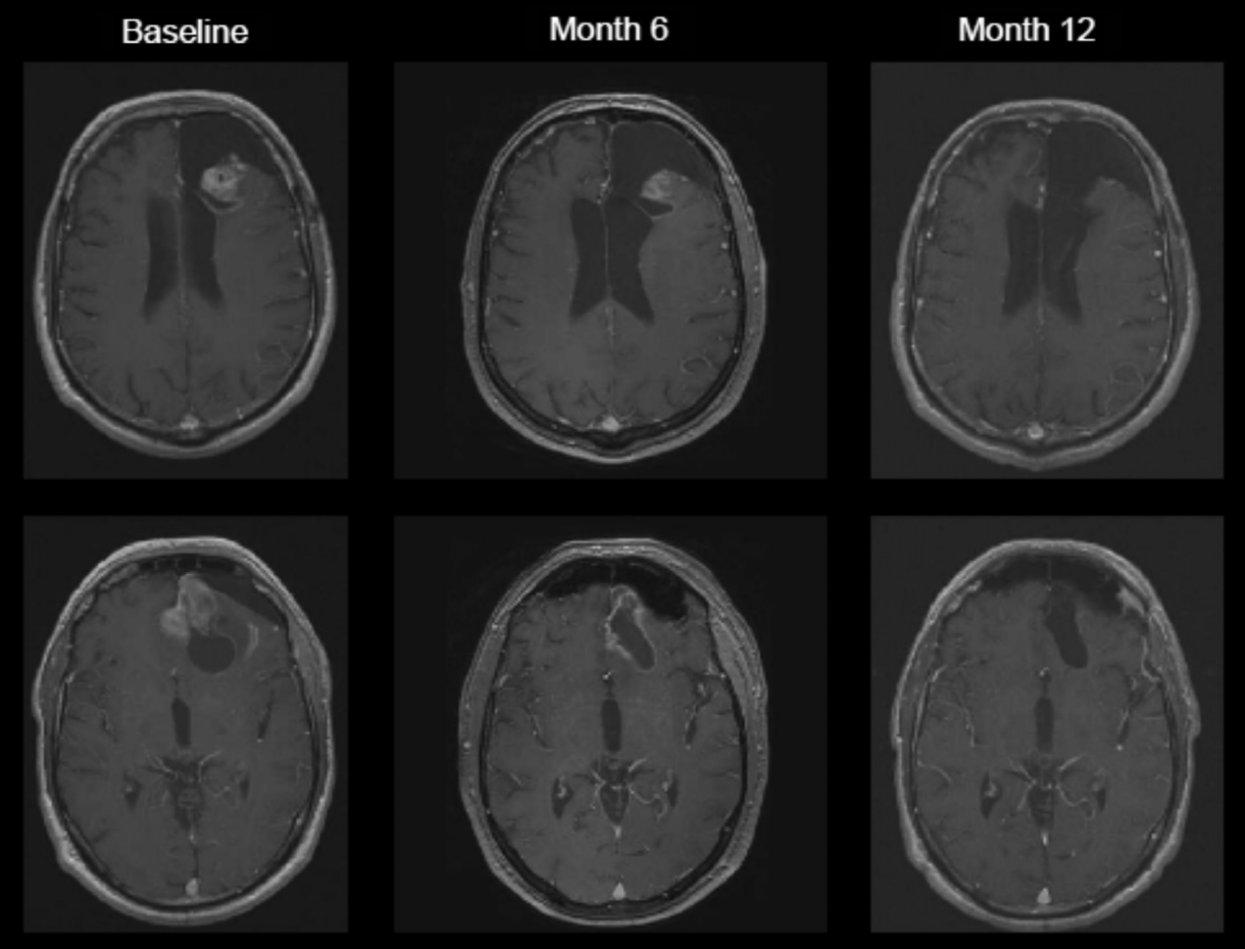
MRI from a complete responder treated with NovoTTF-100A monotherapy. Partial response was noted only after 6 months and complete response was noted after 12 months. MRI, magnetic resonance imaging.

The NovoTTF-100A cohort (*N* = 120) had more responders than the BPC cohort (*N* = 117) (Table 1). The respective median time to response was 8.4 (95% CI 6.9–9.9) months in the NovoTTF-100A responders and 5.8 (95% CI 3.6–8.0) months in the BPC chemotherapy responders (*P* = 0.5755, Figs. 2 and 3). Six of 14 responders (43%) had initial growth of the tumor at 2–24 months while on NovoTTF-100A suggesting a period of tumor pseudoprogression. The median response duration was 7.3 (95% CI 0.0–16.6) and 5.6 (95% CI 3.8–7.5) months, respectively (*P* = 0.0009). These data indicate that, compared to BPC chemotherapy responders, the NovoTTF-100A responders may have had a longer time to response after treatment initiation and, when responded, they had a more durable response.

**Table 1:** Clinical and response characteristics of NovoTTF-100A versus BPC chemotherapy cohorts

	NovoTTF-100A (*N* = 14 of 120)	BPC chemotherapy (*N* = 7 of 117)
Clinical characteristics		
Age, median (range)	54 (36–75)	50 (35–59)
KPS, median (range)	90 (60–100) %	80 (60–100) %
Tumor size, median (range)	13 (2–38) cm^2^	14 (5–44) cm^2^
Prior low-grade glioma	5 (36%)	0 (0%)
Median time from diagnosis	8.3 months	11.8 months
Duration of device usage, median (range)	22 (13–23) h/day	Not applicable
Daily dexamethasone dose, median (range)	0.5 (0.0–12.0) mg	3.0 (0.0–18.0) mg
Response characteristics		
Complete response	3 (3%)	0 (0%)
Partial response	11 (9%)	7 (6%)
Median (95% CI) time to response	8.4 (6.9–9.9) months	5.8 (3.6–8.0) months
	*P* = 0.5755	
Median (95% CI) response duration	7.3 (0.0–16.6) months	5.6 (3.8–7.5) months
	*P* = 0.0009	
Median (95% CI) unadjusted PFS		
Responders	14.8 (11.0–N/A) months	11.3 (9.4–N/A) months
Nonresponders	2.1 (2.0–2.2) months	2.1 (2.0–2.8) months
*χ*^2^	25.5 (*P* < 0.0001)	16.5 (*P* < 0.0001)
Median (95% CI) Simon–Makuch adjusted PFS		
Responders	17.8 (11.5–N/A) months	11.5 (11.4–N/A) months
Nonresponders	10.5 (10.4–10.6) months	7.9 (7.8–8.6) months
*χ*^2^	11.5 (*P* = 0.0007)	5.2 (*P* = 0.0222)
Median (95% CI) OS		
Responders	24.8 (17.5–N/A) months	20.0 (14.5–N/A) months
Nonresponders	6.2 (5.0–7.7) months	6.8 (5.8–8.5) months
*χ*^2^	25.7 (*P* < 0.0001)	5.1 (*P* = 0.0235)

BPC, Best Physician's Choice; χ^2^, chi-squared; CI, confidence interval; N/A, not available; OS, overall survival; PFS, progression-free survival.

**Figure 4:**
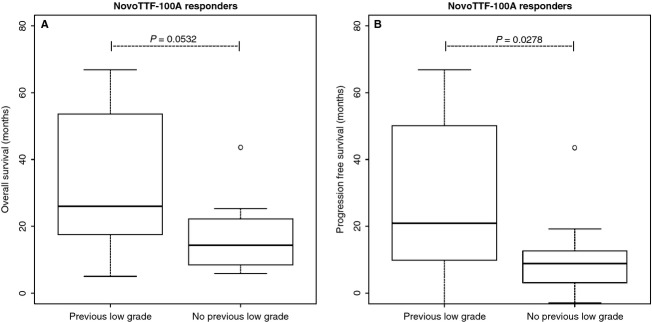
Box-and-whisker plot of OS and PFS of responders in the NovoTTF-100A cohort with and without prior low-grade glioma histology. (A) The median and mean OS was 27.7 and 39.2 (95% CI 19.0–59.4) months among responders with prior low-grade histology compared to 16.6 and 17.0 (95% CI 9.1–24.9) months among those without prior low-grade histology (*P* = 0.0532). (B) The median and mean PFS was significantly prolonged among responders with prior low-grade histology, 18.0 and 34.4 (95% CI 10.6–58.3) months, compared to those without, 5.5 and 10.7 (95% CI 2.2–19.2) months (*P* = 0.0278). The central boxes represent values from first to third quartile (25–75th percentiles). The horizontal line represents the median and the vertical line extends from the minimum to maximum values and shows the presence of outlier. The outlier was not excluded from analysis. OS, overall survival; PFS, progression-free survival; CI, confidence interval.

NovoTTF-100A responders have somewhat different clinical characteristics than BPC responders (Table 1). Notably, five of 14 responders in the NovoTTF-100A cohort, while none of seven responders in the BPC cohort, had prior low-grade histology. Among the NovoTTF-100A responders, there was a trend for increased median and mean OS among those with prior low-grade histology compared to those without, 27.7 and 39.2 (95% CI 19.0–59.4) versus 16.6 and 17.0 (95% CI 9.1–24.9) months, respectively (*P* = 0.0532, Fig. 4A). However, the median and mean PFS was significantly prolonged among those with prior low-grade histology compared to those without, 18.0 and 34.4 (95% CI 10.6–58.3) versus 5.5 and 10.7 (95% CI 2.2–19.2) months, respectively (*P* = 0.0278, Fig. 4B).

**Figure 5:**
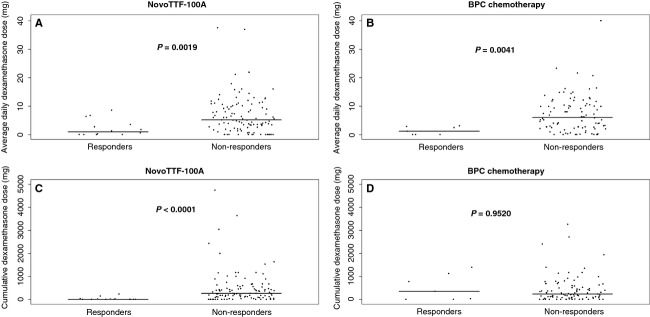
Scatter plot of mean daily dexamethasone and cumulative dexamethasone dose in responders and nonresponders. (A) In the NovoTTF-100A cohort, the respective median and mean daily dexamethasone dose was 1.0 and 2.3 (95% CI 0.8–3.8) mg for responders versus 5.2 and 6.8 (95% CI 5.6–8.1) mg for nonresponders (*P* = 0.0019). (B) In the BPC cohort, the respective median and mean daily dexamethasone dose was 1.2 and 1.4 (95% CI 0.3–2.4) mg for responders versus 6.0 and 7.2 (95% CI 6.0–8.4) mg for nonresponders. (C) In the NovoTTF-100A cohort, the respective median and mean cumulative dexamethasone dose was 7.1 and 35.9 (95% CI N/A–72.5) mg for responders versus 261.7 and 485.6 (95% CI 347.9–623.4) mg for nonresponders (*P* < 0.0001). (D) In the BPC cohort, the respective median and mean cumulative dexamethasone dose was 348.5 and 525.6 (95% CI 96.5–954.7) mg for responders versus 242.3 and 431.0 (95% CI 328.1–533.8) mg for nonresponders (*P* = 0.9520). BPC, Best Physician's Choice; CI, confidence interval; N/A, not available.

Dexamethasone use among responders was also significantly lower than that in nonresponders (Fig. 5). In both NovoTTF-100A and BPC cohorts, responders had a lower daily dexamethasone usage than nonresponders. For the NovoTTF-100A cohort, the respective median and mean daily dexamethasone dose was 1.0 and 2.3 (95% CI 0.8–3.8) mg for responders versus 5.2 and 6.8 (95% CI 5.6–8.1) mg for nonresponders (*P* = 0.0019). For the BPC chemotherapy cohort, the respective median and mean daily dexamethasone dose was 1.2 and 1.4 (95% CI 0.3–2.4) mg for responders versus 6.0 and 7.2 (95% CI 6.0–8.4) mg for nonresponders (*P* = 0.0041). Notably, the cumulative dexamethasone dose was only found to be significantly lower in responders than nonresponders in the NovoTTF-100A cohort but not in the BPC chemotherapy cohort. For the NovoTTF-100A cohort, the respective median and mean cumulative dexamethasone dose was 7.1 and 35.9 (95% CI N/A–72.5) mg for responders versus 261.7 and 485.6 (95% CI 347.9–623.4) mg for nonresponders (*P* < 0.0001). For the BPC chemotherapy cohort, the respective median and mean cumulative dexamethasone dose was 348.5 and 525.6 (95% CI 96.5–954.7) mg for responders versus 242.3 and 431.0 (95% CI 328.1–533.8) mg for nonresponders (*P* = 0.9520). Therefore, in light of the more frequent low-grade histology and the lower cumulative dexamethasone dose, NovoTTF-100A responders may have more favorable genetic and/or epigenetic characteristics. [Correction added on 30th May 2014, after first online publication: “daily” was amended to “cumulative” in the median and mean dexamethasone dose for the BPC chemotherapy cohort. The same has been amended in the legend of Figure 5, section D.]

### Hazard analysis in responders and nonresponders

**Figure 6:**
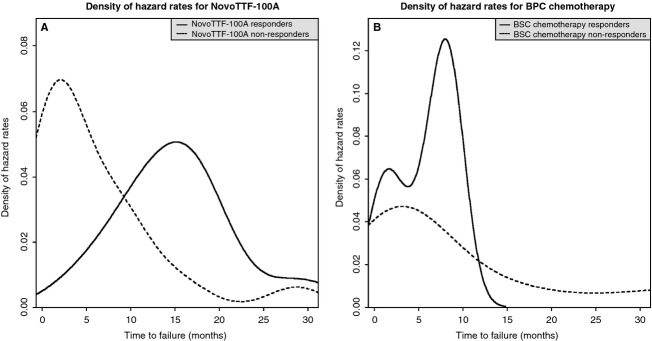
The hazard functions for tumor progression in responders and nonresponders. This is a time-dependent estimate of responders that transition out of response into tumor progression or nonresponders that directly transition into tumor progression. (A) In the NovoTTF-100A cohort, the peak hazard rate for responders is lower than that for nonresponders, 0.051 versus 0.069, respectively, and the time of peak hazard rate is delayed in responders compared to nonresponders, 15.1 versus 1.9 months, respectively. (B) In the BPC chemotherapy cohort, the peak hazard rate for responders is higher than that for nonresponders, 0.125 versus 0.047, respectively, but the time of peak hazard rate is still delayed in responders compared to nonresponders, 8.0 versus 3.1 months, respectively. The higher peak hazard rate for responders could be a result of the small sample size (*N* = 7) and/or most patients go into tumor progression at nearly simultaneous time. BPC, Best Physician's Choice.

The hazard rate of tumor progression initially increased with time, reached a maximum, and then fell to a basal rate in both responders and nonresponders (Fig. 6). However, for NovoTTF-100A responders, the peak hazard rate was lower than that for nonresponders and the time of peak hazard rate was delayed compared to nonresponders. The proportional hazard ratio was 0.1560 (95% CI 0.0698–0.3500, *P* < 0.0001). In contrast, the BPC cohort's hazard rate for responders peaked higher compared to nonresponders, while the time of peak hazard rate is also delayed in responders compared to nonresponders. The proportional hazard ratio was 0.0877 (95% CI 0.0208–0.3700, *P* = 0.0009). The results in both cohorts indicate that responders had a delay in tumor progression, but the higher peak hazard rate in the BPC cohort may be due to their tumor progression at nearly simultaneous time.

### Survival analysis in responders and nonresponders

**Figure 7:**
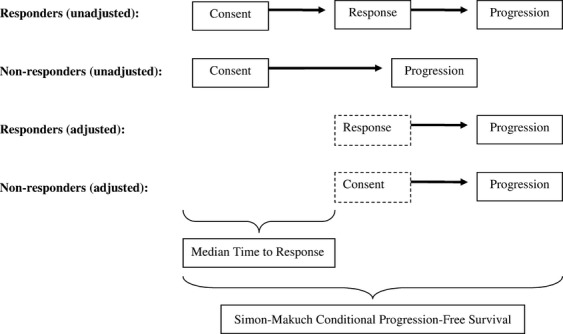
Graphical representation of the adjusted Simon–Makuch conditional PFS. In the intent-to-treat population, PFS is measured from the time of consent until progression or censored event. However, responders pass through a state from consent to response and this time-to-response period introduces a bias in the statistical comparison of responders versus nonresponders, favoring the responder group. To correct this bias, the median time to response is added to both responder and nonresponder groups before comparison of the respective PFS distributions. PFS, progression-free survival.

**Figure 8:**
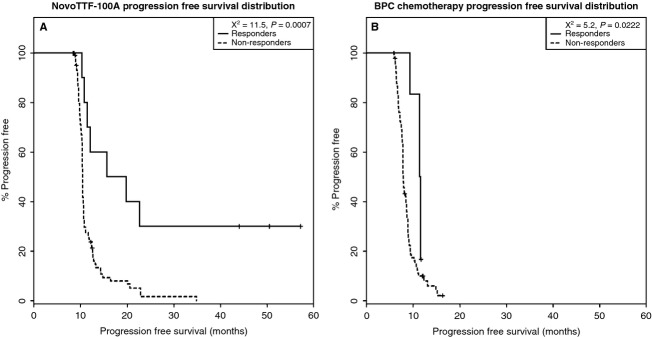
Simon–Makuch conditional PFS distribution. The PFS distribution remains significant after adjustment. (A) In the NovoTTF-100A cohort, the median adjusted PFS is 17.8 (95% CI 11.5–N/A) months for responders and 10.5 (95% CI 10.4–10.6) months for nonresponders. Compared to the value before adjustment, the chi-squared distribution between the two groups remained significant at 11.5 (*P* = 0.0007). (B) In the BPC chemotherapy cohort, the median adjusted PFS is 11.5 (95% CI 11.4–N/A) months for responders and 7.9 (95% CI 7.8–8.6) months for nonresponders. Compared to the value before adjustment, the chi-squared distribution between these two groups also remained significant at 5.2 (*P* = 0.0222). PFS, progression-free survival; CI, confidence interval; N/A, not available; BPS, Best Physician's Choice.

Because responders inherently have a longer period of progression-free state due to the presence of the time-to-response state, adjustment is needed to correct the start time when comparing PFS in responders and nonresponders (Table 1 and Fig. 7). Indeed, in unadjusted analyses for both cohorts, responders had marked prolongation of PFS than nonresponders. To correct this apparent bias, we used the Simon–Makuch adjustment to generate conditional PFS plots 17,18 (Fig. 8). The conditioning time was adjusted based on the Kaplan–Meier estimate of the median time to response in the respective responder groups, or 8.4 months for the NovoTTF-100A versus 5.8 months for the BPC chemotherapy cohort. The adjusted analysis showed that in the NovoTTF-100A cohort, the median Simon–Makuch conditional PFS was 17.8 (95% CI 11.5–N/A) months for responders and 10.5 (95% CI 10.4–10.6) months for nonresponders (*χ*^2^ = 11.5, *P* = 0.0007). In the BPC chemotherapy cohort, the corresponding conditional PFS was 11.5 (95% CI 11.4–N/A) months for responders and 7.9 (95% CI 7.8–8.6) months for nonresponders (*χ*^2^ = 5.2, *P* = 0.0222). After correcting for bias, responders still had a longer adjusted PFS than nonresponder and this difference was unlikely due to chance. This difference was also notably greater in the NovoTTF-100A than the BPC cohort.

**Figure 9:**
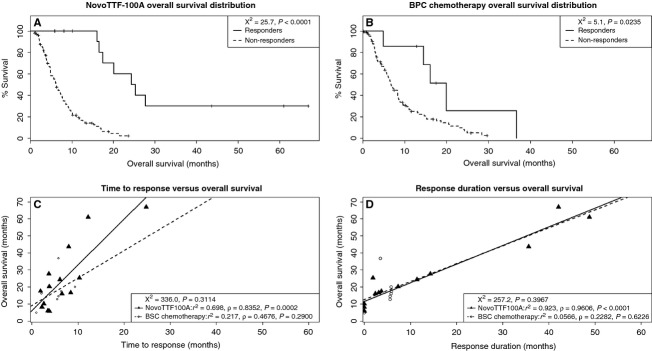
Overall survival distribution between responders and nonresponders. (A) In the NovoTTF-100A cohort, the median OS is 24.8 (95% CI 17.5–N/A) months for responders and 6.2 (95% CI 5.0–7.7) months for nonresponders, and the chi-squared distribution between these two groups is significantly different at 25.7 (*P* < 0.0001). (B) In the BPC chemotherapy cohort, the median OS is 20.0 (95% CI 14.5–N/A) months for responders and 6.8 (95% CI 5.8–8.5) months for nonresponders. The chi-squared distribution between these two groups has a smaller difference at 5.1 (*P* = 0.0235). (C) In the NovoTTF-100A cohort, there is linearity and correlation between OS and time to response (linear regression *r*^2^ = 0.698; Pearson *ρ* = 0.8352, *P* = 0.0002). However, in the BPC cohort, the linearity and correlation are less robust between OS and time to response (linear regression *r*^2^ = 0.217; Pearson *ρ* = 0.4676, *P* = 0.2900). In the chi-squared distribution analysis, there is no statistical difference between OS and time to response (*χ*^2^ = 336.0, *P* = 0.3114), suggesting that OS and time to response are statistically related parameters. (D) In the NovoTTF-100A cohort, there is also linearity and correlation between OS and response duration (linear regression *r*^2^ = 0.923; Pearson *ρ* = 0.9606, *P* < 0.0001). However, in the BPC cohort, there is no linearity or correlation between OS and response duration (linear regression *r*^2^ = 0.0566; Pearson *ρ* = 0.2282, *P* = 0.6226). In the chi-squared distribution analysis, there is no statistical difference between OS and response duration (*χ*^2^ = 257.2, *P* = 0.3967), suggesting that OS and response duration are statistically related parameters. CI, confidence interval; BPS, Best Physician's Choice; N/A, not available; χ^2^, chi-squared.

In OS analysis (Fig. 9), responders treated with either NovoTTF-100A or BPC chemotherapy did better than nonresponders. The median OS was 24.8 (95% CI 17.5–N/A) months for responders and 6.2 (95% CI 5.0–7.7) months for nonresponders in the NovoTTF-100A cohort (*χ*^2^ = 25.7, *P* < 0.0001), while it was 20.0 (95% CI 14.5–N/A) months for responders and 6.8 (95% CI 5.8–8.5) months for nonresponders in the BPC cohort (*χ*^2^ = 5.1, *P* = 0.0235). Because responders were expected to live longer than nonresponders, we next asked whether the time to response or the response duration would correlate with OS in either cohort. In the time to response versus OS analysis, the NovoTTF-100A cohort had a linear regression coefficient of *r*^2^ = 0.698 and a Pearson correlation coefficient of *ρ* = 0.8356 (*P* = 0.0002), suggesting a strong correlation between these two parameters. No such correlation was seen in the BPC cohort, *r*^2^ = 0.217 and *ρ* = 0.4676 (*P* = 0.2900). Similarly, in the response duration versus OS analysis, the NovoTTF-100A cohort had a linear regression coefficient of *r*^2^ = 0.923 and a Pearson correlation coefficient of *ρ* = 0.9608 (*P* < 0.0001). Again, no such correlation was seen in the BPC cohort, *r*^2^ = 0.0566, *ρ* = 0.2282 (*P* = 0.6226). In addition, we used chi-squared distribution analysis to further investigate whether or not there was an association between OS and response. We found no statistical difference between OS and time to response (*χ*^2^ = 336.0, *P* = 0.3114) as well as between OS and response duration (*χ*^2^=257.2, *P* = 0.3967), suggesting that OS and response were related parameters. Together, our data indicated that there was a correlation between response and OS and this effect was predominantly seen in the NovoTTF-100A cohort.

## Discussion

Response is typically a secondary endpoint in cancer clinical trials and, when present, it usually signifies antitumor activity. However, bona fide response may or may not correlate with improved survival for recurrent glioblastoma. A good example is antiangiogenesis drug like bevacizumab, which has a response rate of 25–60% and a 6-month PFS of 45% primarily from nonrandomized, single-arm phase II trials 3. However, it has limited impact on OS 4,5. In contrast, a randomized trial of temozolomide versus procarbazine detected only six of 112 (5.4%) versus six of 113 (5.3%) responders, respectively, but the PFS and OS at 6 months were significantly different, 21% and 8% versus 60% and 44%, respectively 2. This lack of concordance between response and survival probably stems from the low efficacy of single-agent cytotoxic chemotherapy against recurrent glioblastoma 19, which has a plethora of resistant clones endowed with genetic and/or epigenetic derangements. Interestingly, bevacizumab was approved for recurrent glioblastoma based on single-arm, phase II response data while temozolomide was rejected despite positive survival data, indicating that response remains important in the overall efficacy analysis.

Our analysis showed that responders in the two cohorts have different clinical characteristics. First, in the prior phase III trial, 10 of 120 (8%) subjects in the NovoTTF-100A cohort and nine of 117 (8%) subjects in the BPC cohort had prior low-grade histologies 11. However, a significantly higher proportion of NovoTTF-100A responders, five of 14 (36%), had prior low-grade histologies while none of seven (0%) BPC responder had this type of histological characteristics, suggesting that secondary glioblastoma may be more responsive to NovoTTF-100A treatment. Because primary and secondary glioblastomas have different genetic alterations, notably *EGFR* and *MDM2* amplifications together with *p16* deletion in primary glioblastomas and *p53* mutation, *IDH1* mutation and *PDGFR* amplification in secondary glioblastomas, the distinct genetic makeup in these two subtypes of glioblastomas could make secondary glioblastomas more susceptible to NovoTTF-100A treatment 20,21. In the genomic analysis of glioblastoma subtypes, Verhaak et al. 21 found that the majority of secondary glioblastomas have proneural profiles expressing oligodendrocytic development genes such as *PDFGRA* and *OLIG2*. Notably, the proneural subtype is less responsive to concurrent chemotherapy and radiation than the classical, mesenchymal, and neural subtypes 21. Similarly, Ducray et al. reported that there was no significant response to neoadjuvant chemotherapy or radiation alone in the proneural glioblastoma while the mesenchymal and classical subtypes were more likely to respond to radiation and chemotherapy, respectively 22. Therefore, it would be important to determine whether the five NovoTTF-100A responders with previous low-grade histologies also have gene expression profile consistent with the proneural form, as opposed to other subtypes, of glioblastoma. Furthermore, nine of 14 (64%) responders in the NovoTTF-100A cohort had no prior low-grade histology and the response seen in these patients may suggest that there could be additional genetic and/or epigenetic determinants. Second, the daily dexamethasone dose used by both NovoTTF-100A and BPC chemotherapy responders was significantly lower than that used by nonresponders. Indeed, dexamethasone has been associated with profound immunosuppression and increased risk of infection 23. More importantly, patients with lower dexamethasone usage may be more able to mount an anticancer immune response against the glioblastoma 24,25. Our preclinical data indicate that alternating electric fields stress the cytoplasm of dividing tumor cells and that cause the translocation of calreticulin from the endoplasmic reticulum to the surface of cell membrane 25,26. This surface expression of calreticulin could mark the tumor cells for immune destruction. Therefore, this type of antiglioblastoma immune response may be more important for NovoTTF-100A responders than BPC chemotherapy responders, as global immunosuppression by dexamethasone plays a greater role in counteracting the efficacy of NovoTTF-100A than BPC chemotherapy. Taken together, the data on prior low-grade histology and dexamethasone dose suggest potential underlying genetic and/or epigenetics determinants of NovoTTF-100A response.

The response duration, adjusted Simon–Makuch PFS, and OS favor NovoTTF-100A over BPC chemotherapy. First, responders in our NovoTTF-100A cohort behaved similar to a prior analysis by Hess et al. 18, with the hazard rate peaking lower and later than the nonresponders. However, responders in the BPC cohort peaked markedly higher than nonresponders, which could be a result of near-simultaneous tumor progression. Furthermore, the time interval between peak hazard rates of responders and nonresponders in the BPC cohort is narrower than that for the NovoTTF-100A cohort, suggesting that NovoTTF-100A responders had a slightly more favorable tumor progression profile than BPC chemotherapy responders. Second, Simon and Makuch 17,18 introduced a correction by adding the median time to response for every patient in both responder and nonresponder groups and thereby removing the inherent bias in responders when performing survival comparison. Compared to the unadjusted PFS analysis, the Simon–Makuch adjustment showed a smaller but still significant difference in the chi-squared distributions between responders and nonresponders in both NovoTTF-100A and BPC cohorts. Therefore, the difference in PFS between NovoTTF-100A responders and nonresponders remains statistically valid despite the small sample size of responders. Also, this difference is larger in the NovoTTF-100A than the BPC cohort, suggesting that responders possibly experienced a greater efficacy from NovoTTF-100A than responders from BPC chemotherapy. Lastly, we showed an association between survival and response. Our chi-squared analysis cannot reject the null hypothesis that OS versus time to response and OS versus response duration are different in our two cohorts. Notably, Pearson analysis showed that responders to NovoTTF-100A had a stronger correlation than responders to BPC chemotherapy. Hess et al. 16 used Cox proportional hazard analysis of responders to cytotoxic chemotherapies and also found a correlation between OS and response. Together, these data suggest that NovoTTF-100A responders have longer OS and PFS, but a larger sample size is needed to confirm this finding.

There are multiple challenges facing the development of drug therapies for glioblastoma, including parallel and redundant signaling pathways that subserve the growth and proliferation of the tumor, multiple pharmacodynamic targets, the narrow therapeutic index, propensity for the development of resistance, and pharmacokinetic interference from the blood–brain barrier. Therefore, novel treatments that can overcome these challenges are needed. The NovoTTF-100A device fits this profile because it is a locoregional therapy and thereby lacks systemic side effects. Similar to traditional cytotoxic chemotherapies and newer targeted agents, it also interferes with tumor cell mitosis. Specifically, the alternating electric fields emitted by the device block tumor cell progression from metaphase to anaphase, resulting in chromosomal aneuploidy and cytoplasmic stress that ultimately lead to apoptosis, immunogenic cell death, or both 10,26. In this post hoc analysis comparing responders in the NovoTTF-100A and BPC chemotherapy cohorts, we found that secondary glioblastomas and low dexamethasone usage are associated with a higher proportion of NovoTTF-100A responders but not BPC chemotherapy responders. It is notable that in a population-based study performed by Ohgaki et al. 27, secondary glioblastomas appeared to have a slower rate of decline in survival than primary glioblastomas. We speculate that patients whose glioblastomas arose from prior low-grade gliomas may have a slower growth rate than those from primary glioblastomas. When treated with NovoTTF-100A, this slower rate of tumor progression might allow enough time for the efficacy of TTFields to emerge because it may take multiple mitotic cycles to reduce the number of tumor cells and the size of the glioblastoma. This slower rate of growth may not matter as much for BPC chemotherapies due to their direct genomic toxicity. Furthermore, the cytoplasmic stress induced by the alternating electric fields also marks the tumor cells for immunological destruction and clearance 26. Therefore, removal of immunosuppression in the patient, such as reducing or discontinuing dexamethasone usage, would have a greater effect on those receiving NovoTTF-100A treatment than BPC chemotherapy. Taken together, a possible slower rate of tumor growth in secondary glioblastomas and a reduction in immunosuppression caused by dexamethasone may be the underlying mechanisms for the higher number of responders observed in the NovoTTF-100A cohort.

Future clinical trials on the NovoTTF-100A device must include stratification of potential predictive factors of response that include both genetic and epigenetic determinants. It is important to note that the genetic makeup of secondary glioblastomas is different from those of primary glioblastomas and these differences may determine whether or not a glioblastoma responds to a specific therapy. Therefore, genetic profiling of the tumor among patients enrolling into future NovoTTF-100A clinical trials would greatly facilitate the identification of those who are likely, as well as others who are unlikely, to respond to treatment. Furthermore, future trials may also need to include immune modulator that may augment the immunological effect of alternating electric fields. Such concerted approach to treatment will hopefully increase the response rate and efficacy of NovoTTF-100A against glioblastoma.
